# Preliminary Post-Mortem COVID-19 Evidence of Endothelial Injury and Factor VIII Hyperexpression

**DOI:** 10.3390/diagnostics10080575

**Published:** 2020-08-09

**Authors:** Luigi Cipolloni, Francesco Sessa, Giuseppe Bertozzi, Benedetta Baldari, Santina Cantatore, Roberto Testi, Stefano D’Errico, Giulio Di Mizio, Alessio Asmundo, Sergio Castorina, Monica Salerno, Cristoforo Pomara

**Affiliations:** 1 Department of Clinical and Experimental Medicine, University of Foggia, 71122 Foggia, Italy; luigi.cipolloni@unifg.it (L.C.); francesco.sessa@unifg.it (F.S.); gius.brt@gmail.com (G.B.); santina.cantatore@unifg.it (S.C.); 2 Department of Anatomical, Histological, Forensic and Orthopedic Sciences, Sapienza University of Rome, 00186 Rome, Italy; benedetta.baldari@uniroma1.it; 3 Dipartimento di Prevenzione ASL, 10121 Torino (To), Italy; roberto.testi@unito.it; 4 Department of Medical, Surgical and Health Sciences, University of Trieste, 34100 Trieste, Italy; stefanoderrico@hotmail.com; 5 Department of Law, Forensic Medicine, Magna Graecia University of Catanzaro, 88100 Catanzaro, Italy; giulio.dimizio@unicz.it; 6 Dipartimento di Scienze Biomediche, Odontoiatriche e Delle Immagini Morfologiche e Funzionali, Sezione di Medicina Legale, Università di Messina, 98122 Messina, Italy; alessio.asmundo@unime.it; 7 Anatomy, Department of Medical and Surgical Sciences and Advanced Technologies G.F. Ingrassia, University of Catania, 95121 Catania, Italy; sergio.castorina@unict.it; 8 Department of Medical, Surgical and Advanced Technologies “G.F. Ingrassia”, University of Catania, 95121 Catania, Italy; monica.salerno@unict.it

**Keywords:** forensic science, forensic pathology, COVID-19, autopsy, immunohistochemistry, post-mortem examination, COVID-19 diagnostic

## Abstract

(1) Background: The current outbreak of COVID-19 infection is an ongoing challenge and a major threat to public health that requires surveillance, prompt diagnosis, as well as research efforts to understand the viral pathogenesis. Despite this, to date, very few studies have been performed concerning autoptic specimens. Therefore, this study aimed: (i) to reiterate the importance of the autoptic examination, the only method able to precisely define the cause of death; (ii) to provide a complete post-mortem histological and immunohistochemical investigation pattern capable of diagnosing death from COVID-19 infection. (2) Methods: In this paper, the lung examination of two subjects who died from COVID-19 are discussed, comparing the obtained data with those of the control, a newborn who died from pneumonia in the same pandemic period. (3) Results: The results of the present study suggest that COVID-19 infection can cause different forms of acute respiratory distress syndrome (ARDS), due to diffuse alveolar damage and diffuse endothelial damage. Nevertheless, different patterns of cellular and cytokine expression are associated with anti-COVID-19 antibody positivity, compared to the control case. Moreover, in both case studies, it is interesting to note that COVID-19, ACE2 and FVIII positivity was detected in the same fields. (4) Conclusions: COVID-19 infection has been initially classified as exclusively interstitial pneumonia with varying degrees of severity. Subsequently, vascular biomarkers showed that it can also be considered a vascular disease. The data on Factor VIII discussed in this paper, although preliminary and limited in number, seem to suggest that the thrombogenicity of Sars-CoV2 infection might be linked to widespread endothelial damage. In this way, it would be very important to investigate the pro-coagulative substrate both in all subjects who died and in COVID-19 survivors. This is because it may be hypothesized that the different patterns with which the pathology is expressed could depend on different individual susceptibility to infection or a different personal genetic-clinical background. In light of these findings, it would be important to perform more post-mortem investigations in order to clarify all aspects of the vascular hypothesis in the COVID-19 infection.

## 1. Introduction

The current outbreak of COVID-19 infection, which started in Wuhan, Hubei province, China, in December 2019 is an ongoing challenge and a major threat to public health that requires surveillance, prompt diagnosis as well as research efforts to understand this emerging pathogen and to develop effective countermeasures [1,2]. 

Despite the increasing number of published studies on COVID-19, there is an evident lack, in all examined studies, of a well-defined pathophysiology of death among patients who died with or from COVID-19 infection. Several developed countries, such as Italy, did not allow autopsies to be performed in order to contain the risk of infection. This incomprehensive choice can be considered a missed opportunity and a real lockdown of science [3]. 

Therefore, this study aimed: (i) to reiterate the importance of the autoptic examination, the only method able to precisely define the cause of death; (ii) to satisfy the double meaning of the word diagnosis: determination of the nature of a disease and the knowledge of its pathway to distinguish one disease from another through the use of a complete post-mortem histological and immunohistochemical investigation capable of diagnosing death from COVID-19 infection and better understanding its mechanism. Indeed, defining the physiopathological pathway, it will be possible to define serological or tissue biomarkers in order to quickly make a diagnosis in suspected patients, improving their outcome. 

## 2. Materials and Methods 

### 2.1. Cases

Two cases of COVID-19 subjects were collected from among the autopsies performed by the Institute of Legal Medicine of the University of Foggia from January 2020 to April 2020. All procedures were performed in accordance with the Declaration of Helsinki and were approved by the Scientific Committee of the University of Foggia (code: 07_04_2020_RH). The laboratory analyses were performed by the Institutes of Legal Medicine of the Universities of Foggia and Catania. A case of a subject who died of pneumonia in the same period was analyzed as control (Table 1). 

**Case 1:** a 42-year-old male who died from COVID-19 infection. In the middle of February, he had a medical consultation for several respiratory signs. Blood pressure was normal, cardiac activity was regular, no noise was detected at lung auscultation but abdominal pain was observed. Taking into account his personal history of alcoholism, pancreatitis was suspected; treatment with a non-steroidal anti-inflammatory drug (NSAID) was prescribed. Two days later the patient was found dead in his apartment. No other clinical information was discovered after talking to the general practitioner.

**Case 2:** a 70-year-old male who died from COVID-19 infection. At the end of February, the subject visited his son who lived in Bergamo, the epicenter of the COVID-19 pandemic infection in Italy. One week later, he had a medical consultation for several common signs of a respiratory flu, such as fever, cough, asthenia without dyspnea, and minor respiratory symptoms. At the medical consultation, blood pressure was normal, there was regular cardiac activity, no noise at lung auscultation, no neck stiffness, and no sensory-motor deficit. He was treated with penicillin, but two days later there was a sudden worsening of existing symptoms. Particularly, respiratory failure and disorientation were observed. He was transferred to the emergency department of a nearby hospital, but a few minutes later, he died despite the fact that all resuscitation procedures were applied. Previous clinical history was nil. 

**Control case:** a 1-month-old baby boy who died of pulmonary infection not due to COVID-19 related. He was a premature newborn (36 weeks + 1 day), affected by bicuspid aorta and palate malformation. When he was 3 days old, he had a cardio-respiratory depression with brain injury. After a 16 day-hospitalization, he was dismissed. At the end of April, he started demonstrating cyanosis and, in a few minutes, a cardio-respiratory failure occurred. Epistaxis and mouth bleed were observed. He was transferred to the emergency department of a nearby hospital, but after 30 min, he died despite the fact that all resuscitation procedures were applied. Even if this control presents many differences compared to the tested cases, this case was selected because during the pandemic period each case is treated as suspected COVID-19 infection, particularly cases with a lung infection.

Permission was given by the Ethics Committee of the Riuniti Hospital of Foggia to use the patients’ personal and sensitive data for scientific research related to the disease.

### 2.2. Autopsy and Macroscopical Investigation

A full autopsy was performed in all cases. All organs were macroscopically examined: dimensions, weight and section were reported. Due to the special infection-control precaution of handling deceased subjects with COVID-19, the post-mortem examination was performed in a designated autopsy room, following the World Health Organization recommendations [4]. The lung findings are reported in this paper.

### 2.3. Histological Investigation

All formalin-fixed, paraffin-embedded (FFPE) tissue specimens were sampled. The histopathology and pathological characteristics were analyzed by hematoxylin and eosin (H&E) staining following the standard protocol [5]. Moreover, additional slides of lung sections were stained with modified Masson’s trichrome stain to determine any increase in collagen fibers [6] staining the collagen fibers blue, the nuclei black, while the background was red.

Sections were mounted and the results were viewed using a light microscope (Leica DM4000B, Leica, Cambridge, UK).

### 2.4. Immunohistochemical Investigation

The FFPE tissues were cut into 2–3 μm sections and collected on poly-l-lysine-charged glass slides. The paraffin sections were mounted on slides covered with 3-aminopropyltriethoxysilane (Fluka, Buchs, Switzerland). Pre-treatment was necessary to facilitate antigen retrieval and to increase membrane permeability to antibodies. The primary antibody was applied in a 1:500 ratio for all antibodies and incubated for 120 min at 20 °C. The detection system used was the LSAB+ kit (Dako, Copenhagen, Denmark), a refined avidin-biotin technique in which a biotinylated secondary antibody reacts with several peroxidase-conjugated streptavidin molecules. The sections were counterstained with Mayer’s hematoxylin, dehydrated, cover slipped and observed under a Leica DM4000B optical microscope (Leica, Cambridge, UK).

The following anti-human antibodies for the immunohistochemistry investigation were used: -anti-COVID nucleocapsid antibody (anti Coronavirus -FIPV3-70 Santa Cruz Biotechnology, Inc., Dallas, TX, USA); -anti-CD-4 (Santa Cruz Biotechnology, Inc., Dallas, TX, USA); -anti-CD-8 (Santa Cruz Biotechnology, Inc., Dallas, TX, USA); -anti-CD-20 (Santa Cruz Biotechnology, Inc., Dallas, TX, USA); -anti-CD-68 (Santa Cruz Biotechnology, Inc., Dallas, TX, USA); -anti-CD-79 (Santa Cruz Biotechnology, Inc., Dallas, TX, USA); -anti factor VIII (to evaluate the endothelial cell control of thrombosis activation); -pro-inflammatory cytokines such as TNFα, IL-6, and ACE2.

A qualitative scoring was assigned for each sampled tissue. As previously described, scientists may use qualitative scoring systems to interpret received data: usually the force of immunohistochemical staining in different investigated areas. Score ranks usually lie in a range from “negative” (mostly marked as “−”) to “positive”, which may be indicated with a different number of “+” depending on how many other categories lay between these parameters [7,8,9]. In the present study, the evaluation of each immunohistochemical staining was performed, indicating the different force of immunohistochemical expression with the following legend: “negative” (−), “very weak” (+/−), “weak” (+), “moderate” (++), “strong” (+++), “very strong” (++++). Each tissue was evaluated blind by ten different pathologists. Finally, the mean value is summarized in Table 1.

## 3. Results

The gross lung investigation, histopathological evaluation (H&E and Masson’s trichrome stain) and immunohistochemical staining were performed.

### 3.1. Gross Findings

**Figure 1:**
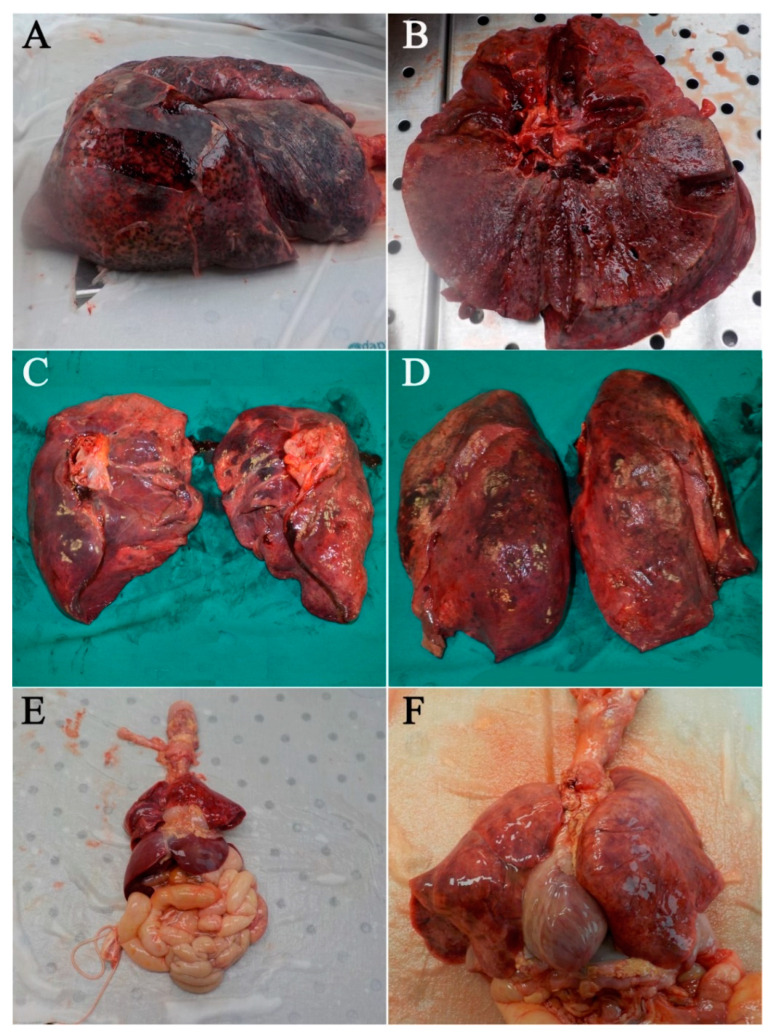
Lungs of Case 1 (**A**,**B**): the left lung had a greater volume while the right lung showed normal volume and shape (**A**). The lungs showed bilateral pulmonary edema and the left one presented patches of dark hemorrhage (**B**). Lungs of Case 2 (**C**,**D**). Lungs of the Control Case (**E**,**F**).

**Figure 2:**
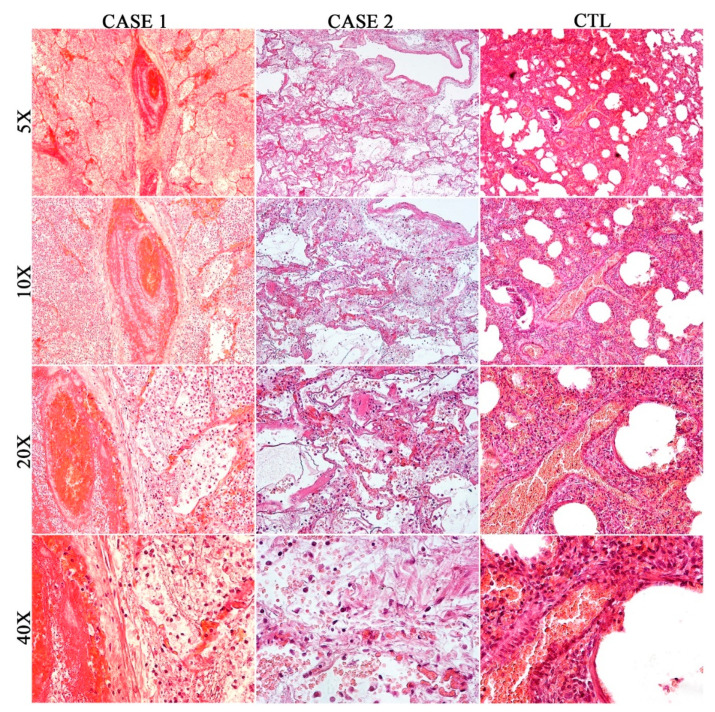
H&E staining: left lung of Case 1 (cm 26 × 20 × 8 and g 1465) showing unilateral diffuse alveolar damage with a focus on a microthrombus and surrounding lung tissue characterized by severe organizing pneumonia. Sample from lungs of Case 2 (left: cm 26.5 × 17 × 2.5 and g 1000; right: cm 26 × 17.5 × 3 and g 1040): showing bilateral diffuse alveolar damage with alveolar protein and fibrinous exudate, associated with wall thickening and abundant macrophagic alveolar infiltrate. Sample from lungs of CTL (left: 7 × 5 × 1.5 cm and 31 g; right: 6 × 5.5 × 2.5 cm and 29 g): showing bilateral interstitial pneumonia, consisting mainly of monocytic-macrophage cells, plasma cells, and rare granulocytes.

The gross examination of the lungs showed heavy lungs and usual lobes and fissures, in both Cases 1 (Figure 1A,B) and 2 (Figure 1C,D). Other common aspects were: pulmonary arteries at the hilum of each lung were free of thromboembolic formations; while bronchi dissection revealed pink froth in the airways; the parenchyma was diffusely edematous and firm, in both cases. On the other hand, the left lung from Case 1 (in detail: the left lung measured cm 26 × 20 × 8 and weighed g 1465; right lung measured cm 25 × 19 × 7.5 and weighed g 806) showed macroscopic regions of dark, reddish color, focally demarked from the surrounding parenchyma, both pleural and intraparenchymal. However, in Case 2, at the lung examination (the left lung measured cm 26.5 × 17 × 2.5 and weighed g 1000; right lung measured cm 26 × 17.5 × 3 and weighed g 1040) small whitish fibrotic areas were found.

Lungs, collected from the Control case (Figure 1E,F), appeared normal in shape, dimensions, and consistency (the left lung dimensions were 7 × 5 × 1.5 cm and weighed 31 g; right lung dimensions were 6 × 5.5 × 2.5 cm and weighed 29 g) compatible with the age of development. Nothing to report about the external surfaces after cutting them.

### 3.2. Histological Findings

**Figure 3:**
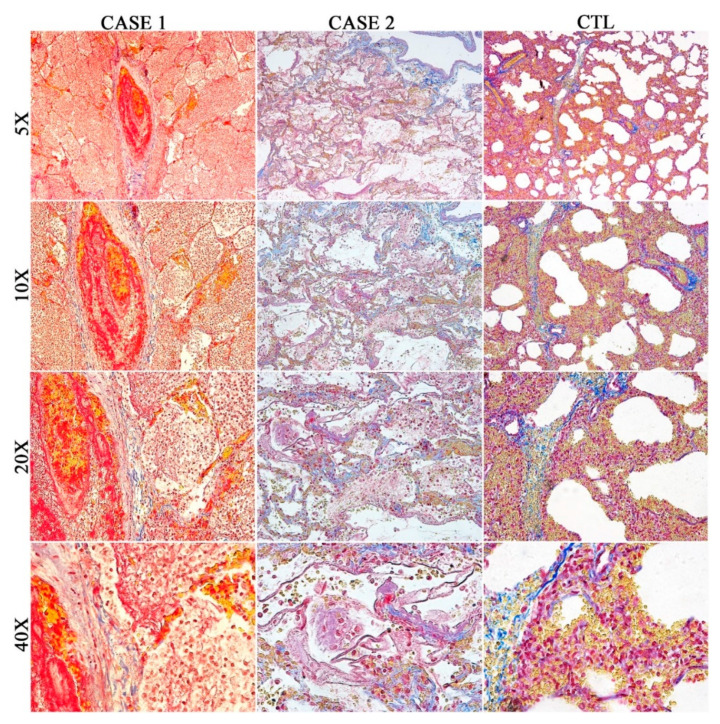
Masson’s trichrome stain: left lung of Case 1 confirmed the microthrombus and there was the presence of a modest deposit of perivasal fibrin in blue. Sample from lungs of Case 2 showed the presence of hyaline membranes, almost ubiquitous. Sample from lungs of CTL: Masson’s trichrome stain confirmed the H&E findings.

In Case 1, on histological examination (H&E staining), the left lung showed severe organizing pneumonia, which, in some fields, assumed a hemorrhagic appearance. The infiltrating cells, mainly composed of plasma cells and macrophages, and few lymphocytes, completely filled the alveolar spaces in some fields. Moreover, extensive edema of the alveolar spaces was described, and the septa appeared thickened with the proliferation of fibrous connective tissue and abundant leukocytes (Figure 2). The study of the vessels showed fibrin deposition within the lumen and walls. Moreover, to detect connective tissue, in particular mature collagen fibers, Masson’s trichrome staining was performed on all lung sections, highlighting the presence of a modest deposit of perivasal fibrin in blue (Figure 3). A few hyaline membranes were noticed. Presence of stratification with concentric layers of fibrin inside a small vessel that retains a great abundance of well distinguishable erythrocytes (microthrombus and intussusceptive angiogenesis) [10].

The microscopic study of Case 2 documented alterations of normal tissue cytoarchitecture, affecting both lungs. The H&E staining showed varying degrees of the proliferative phase of diffuse alveolar damage (DAD) (Figure 2).

Evident alveolar protein and fibrinous exudate were associated with wall thickening, proliferating interstitial fibroblasts and hyperplasia of type II pneumocytes. Some areas showed abundant macrophage alveolar infiltrate. In one field, there was a microthrombus inside the septal capillary.

Finally, an extensive extracellular fibrin deposition was detected by means of Masson’s trichrome stain (Figure 3), which highlighted the presence of hyaline membranes that were almost ubiquitous (Figure 3).

The main findings of the not due to COVID-19 pneumonia, Control case, are summarized in Figure 2 and Figure 3. Both lungs showed regular thickness pleura. In many fields, there was considerable thickening of the septal structures due to the presence of amorphous eosinophilic material as well as abundant cellularity consisting mainly of monocytic-macrophage cells, plasma cells, and rare granulocytes. The air spaces appeared, in some fields, atelectatic, and, in others, showed amorphous eosinophilic material inside. In rare fields, the alveolar spaces appeared to contain poor cellularity consisting mainly of macrophages.

### 3.3. Immunohistochemical Findings

**Figure 4:**
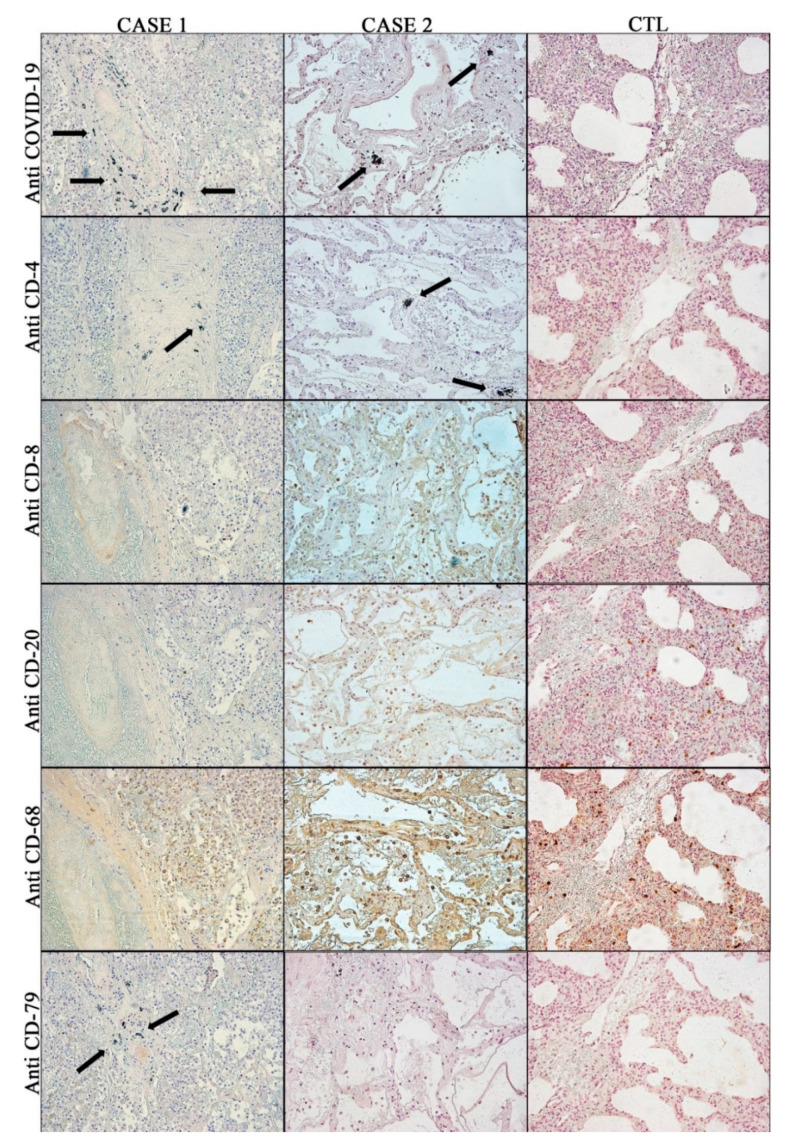
Immunohistochemical findings (20x): arrows show focal positivity; in the other pictures the positivity for each antibody appears diffuse. In particular, very strong positivity for the anti-COVID-19 Ab in both Cases 1 and 2; weak positivity for the anti-CD-4 Ab in both Cases 1 and 2; CD-8+ lymphocytes were located predominantly in the interstitial spaces and around larger bronchioles in both Cases 1 and 2; Anti-CD-20 and anti-CD-68 strong positivity in Cases 1 and 2 confirm the histochemical microscopic data of the presence of plasma cells and macrophages; anti CD-79 Ab shows the highest positivity in Case 1, being completely negative in Case 2. The Control Case showed the picture of interstitial pneumonia with a prevalent macrophagic and lymphoplasmatic cellularity: COVID-19 negative; Ab-anti-CD-20 and Ab-anti-CD-68 positive.

The immunohistochemical findings are summarized in Table 2:

As reported in Figure 4, the samples of both Cases 1 and 2 were positive for the immunohistochemical stain anti-COVID-19, confirming the positivity for a viral infection. For this kind of antibody, the positivity was higher in Case 1, compared to Case 2. The Control case confirmed the clinical data and was negative.

As regards the immunohistochemical cell characterization, the greatest positivity in both Cases 1 and 2 was detected with the use of anti-CD-20 and anti-CD-68 antibodies, confirming the histochemical microscopic data of the presence of plasma cells and macrophages (whose positivity was the highest in both Cases 1 and 2) in the leukocyte infiltrate.

The presence of CD-4+ and CD-8+ T cells was evaluated by immunohistochemistry: the specimens of both Cases 1 and 2 were positive for these reactions. The lymphocytic infiltrate was a mixture of CD-4+ and CD-8+ lymphocytes (Figure 4), located predominantly in the interstitial spaces and around larger bronchioles.

The main findings of no COVID-19 pneumonia, Control case, are summarized in Figure 4, confirming the picture of an interstitial pneumonia with a prevalent macrophagic and lymphoplasmatic cellularity.

**Figure 5:**
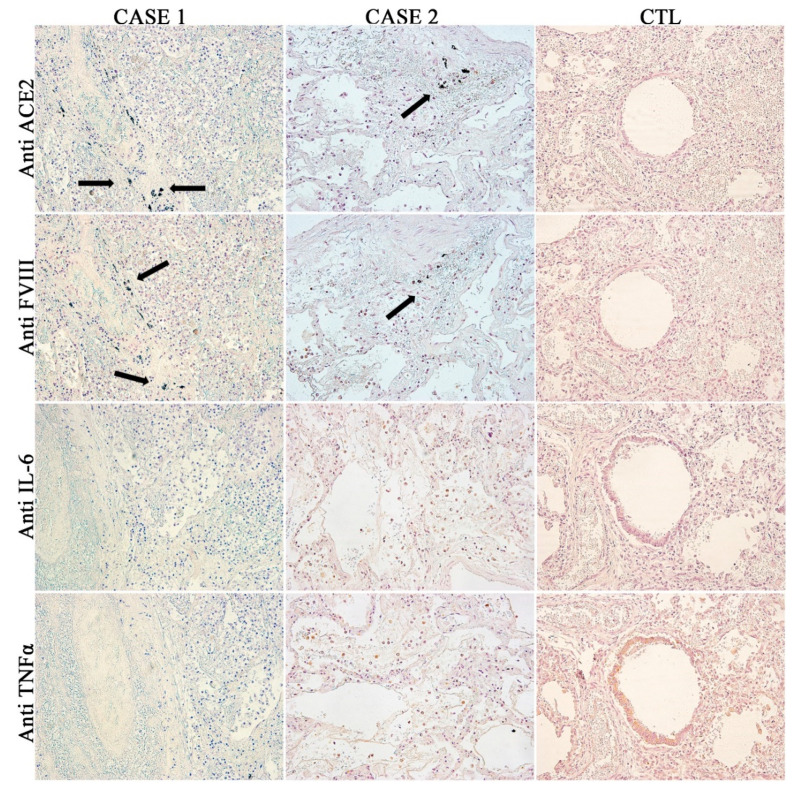
Immunohistochemical findings (20×): Ab-anti-ACE2 showing high positivity in both Cases 1 and 2, instead Ab-anti-FVIII shows the highest positivity in Case 1, being completely negative in the Control case. Moreover, the Ab-anti-ACE2, Ab-anti-FVIII, and Ab anti-COVID-19 in Figure 4, located in the same site. Ab-anti-IL6 and Ab-anti-TNFα was positive in all samples.

Finally, the anti-factor VIII antibody reaction (to evaluate the endothelial cell control of thrombosis activation), pro-inflammatory cytokine antibodies (TNFα, IL-6), and ACE2 immunohistochemical test were performed on the same samples and are summarized in Figure 5.

## 4. Discussion

This study reports two cases of death from COVID-19, comparing the obtained data with those of a Control case, a newborn who died from pneumonia not due to COVID-19. Considering that COVID-19 infection is new, the main findings of the present study confirm that autopsy represents a very important tool to clarify the mechanisms of infection [11,12,13,14,15,16,17].

The gold standard method to identify the SARS-Cov2 infection is polymerase chain reaction (rRT-PCR) performed on different respiratory samples, such as nose and throat swabs [18,19]. It is interesting to note that in Case 1 the post-mortem nasopharyngeal and oropharyngeal flocked swabs were negative for the laboratory diagnosis of COVID-19, which were made using (real-time) reverse transcription-rRT-PCR. On the other hand, in Case 2 the post-mortem nasopharyngeal swab was positive. These results confirmed the analytical vulnerabilities of the kit used for the laboratory diagnosis of COVID-19. As described by Lippi et al. [20] and remarked on in a recent review [21], there are several criticisms of rRT-PCR that could negatively influence the results. Particularly, in Case 1, the subject was in the first phase of the infection (he died about 2 days later), and it may be conjectured that the viral load in his nose and throat was under the rRT-PCR threshold value, thus negative. For these reasons, in all post-mortem examinations, both performed for juridical and no juridical purposes, both fresh and fixed samples of all organs and biological fluids should be collected to perform all necessary investigations (such as histological and immunohistochemical analyses, molecular and serological tests) in order to ascertain the positivity or negativity of the subject, independently from the swab result. Another important criticism of this technique is related to the sampling phase: according to recent estimates, false negative results due to inadequate sampling obtained with rRT-qPCR are more common than initially thought [20]. This limitation could be higher during post-mortem sampling, considering that in several cases, the post-mortem interval from death to swab is unknown.

Analyzing the histological and immunohistochemical findings the results of the present study confirmed that COVID-19 infection generates similar lung injuries to those observed in SARS-CoV (Severe Acute Respiratory Syndrome coronavirus infection) [22] and MERS-CoV (Middle East respiratory syndrome coronavirus infection) [23]. As previously described [24,25], COVID-19 infection can cause different forms of acute respiratory distress syndrome (ARDS), due to diffuse alveolar damage and diffuse endothelial damage.

Regarding the tissue pattern, this study describes two different phenotypes. The first case dealt with a severe unilateral pneumonia organization in a young subject, with leukocyte infiltration mainly represented by plasma cells and macrophages, which appeared very similar to Magro-atypical ARDS [16]. The second case, on the other hand, showed bilateral alterations consisting of hyaline membrane, type II pneumocyte hyperplasia, and mild interstitial thickening affecting an older person, as reported by different authors [16,17,26,27,28].

Evaluating the immunohistochemical investigation results, both Cases 1 and 2 showed a positive reaction to the specific Ab anti-COVID-19, demonstrating the presence of the SARS-CoV-2 nucleoprotein (COVID-19), while the Control case was negative at the immunohistochemical test to Ab anti-COVID-19. As previously discussed in Case 1, the rRT-PCR for COVID-19 was negative: our findings confirm that the virus physiopathology starts from the lower airways, reaching the upper airways later. For these reasons, in agreement with Sessa et al. [21] it is important to follow a specific workflow in autopsy performed during the COVID-19 outbreak.

Moreover, although CD-8+ T cells and CD-4+ T cells are thought to play a major role in effective anti-infection responses, in both Cases 1 and 2, we found a similar positive response for CD-8, while the presence of CD-4 was higher in Case 1 compared to Case 2. Magro et al. [29] described the same findings in the examined lung sampled from 5 patients with COVID-19 infection and severe respiratory failure. Moreover, the investigation to evaluate the presence of CD-20, CD-68 and CD-79 had not been reported previously. Even if the immunohistochemical analysis showed similar results referring to the presence of CD-20 and CD-68 cells, the scenario is completely different evaluating the presence of plasma cells. This kind of cells was evaluated using CD-79a antibodies: even if not well understood, this kind of investigation could be used in order to likely distinguish between the two patterns of the same infection. Moreover, in both cases there were the positivity for TNFα and IL-6 antibodies confirming the activation of a specific immune pathway in response to the COVID-19 infection. Recent reports about the pathological pathway of COVID-19 suggested that one of the most important consequences of this infection is the cytokine storm syndrome [30,31,32] that could be strictly linked to coagulopathy, generating acute pulmonary embolism caused by in-situ thrombosis [33,34,35].

Furthermore, the angiotensin-converting enzyme 2 (ACE2) antibody reaction was applied to the lung samples of both cases. It is well known that ACE2, and putatively also sialic acids, represents the “door” by which COVID-19 enters endothelial cells and pericytes; ACE2 receptors are ubiquitous, not only present in the endothelial cells of the alveolar membrane [36,37]. It is very interesting to note that the same areas that were positive for anti-COVID-19 were immunoreactive for ACE2 antibodies, strongly supporting this hypothesis [38,39].

Finally, for the first time, the immunoreaction for factor VIII was applied, based on the fact that particular pulmonary endothelial cells are a source of FVIII synthesis [40,41]. Interestingly, as summarized in Table 2, it is possible to distinguish two different pictures at the lung level in COVID-19 patients. Particularly, in Case 1 we had a marked positivity, suggesting an interesting point of view about the COVID-19 infection: in several cases, it is possible to find a pro-thrombotic scenario. Considering the thrombotic aspect of 21 COVID-19 subjects from the Menter et al. study [42], the cause of death was attributed to the widespread alveolar damage in the exudative phase with capillary congestion accompanied by microthrombi despite anticoagulant therapy. Other morphological substrates, only in some cases, were superimposed bronchopneumonia, pulmonary embolism, alveolar hemorrhage and vasculitis [42]. Varga et al. [43] reported a widespread endothelial inflammation in 3 positive cases of COVID-19, including 1 case with the presence of lymphocytic endothelitis in lung, heart, kidney and liver samples, assuming that endothelial dysfunction is the main determinant of COVID-19 disease, which induces vasoconstriction with subsequent organ ischemia, inflammation, edema and pro-coagulant state. In support of the idea of an increased pro-coagulation state, Dolhnikoff et al. [44] reported in 8 out of 10 positive cases of COVID-19 the presence of small fibrinous thrombi in the pulmonary arterioles in areas of both damaged and more conserved lung parenchyma and a large number of megakaryocytes in the capillaries. A recent confirmation of this pathophysiological mechanism is described in the paper of Ackermann et al. [10]; in this study widespread alveolar, intra-alveolar fibrin deposition and fibrin thrombus were documented in pulmonary arterioles, without complete luminal obstruction and, in some cases, associated with intussusceptive angiogenesis. In the same study, 69 angiogenesis-related genes were revealed to be differentially regulated during the COVID-19 infection. Further confirmation of the involvement of coagulopathy and thrombosis models has recently been reported in the study by Carsana et al. [45]: these authors described the formation of fibrin microthrombi in small arterial vessels (<1 mm in diameter) in the context of areas of diffuse alveolar damage associated with diffuse endothelial damage. Lung tissue also had changes in exudative and early or intermediate DAD, namely pneumocyte necrosis (in all cases), hyaline membrane (in 33 cases), interstitial and intra-alveolar edema (in 37 cases), type 2 pneumocytic hyperplasia (in all cases). These results are associated with an inflammatory infiltrate, consisting of alveolar macrophages and interstitial lymphocytes. The ante-mortem involvement of both established DAD and disseminated intravascular coagulation (DIC) models may explain the serious hypoxemia that characterizes ARDS in subjects with COVID-19 [46]. Finally, the FVIII-von Willebrand Factor (vWF) complex is another prothrombotic unit responsible for the adhesion of platelets to subendothelial structures, released by endothelial cells when injured, in association with severe endothelial dysfunction; thus, it has been suggested as a biomarker of endothelial dysfunction, which is largely involved in COVID-19 pathogenesis [47,48,49,50,51,52]. Moreover, it is interesting that COVID-19, ACE, and FVIII positivity were collected in the same fields.

The main limitation of this paper is related to the number of cases: limiting the observation to only two cases excludes a wide spectrum of diseases. However, it has to be remembered that this study took place in a period in which post-mortem examinations were not recommended in Italy [3]. Furthermore, the choice of the Control case may seem too different in age from the two cases. However, the selection was made by identifying a case of diagnosed pneumonia that occurred in the same historical period as the two cases and certainly negative for COVID-19 infection. The presence of a few cases did not allow a statistical analysis. Therefore, it can be considered a pilot research whose case series will be expanded in subsequent studies. On the contrary, the strength of this paper is related to the application, for the first time, of the complete immunohistochemical reaction on lung samples, examining several critical biomarkers such as factor VIII (to evaluate endothelial cell control of thrombosis activation) and pro-inflammatory cytokines such as TNFα, IL-6. Further studies should investigate other inflammation biomarkers that could be modulated by COVID-19 infection.

## 5. Conclusions

COVID-19 infection was initially classified as exclusively interstitial pneumonia with varying degrees of severity. Subsequently, vascular biomarkers showed that it can also be considered a vascular disease. Particularly, several biomarkers such as D-dimer and troponin can be very useful suggesting earlier and more aggressive interventions and treatments in order to avoid and/or minimize arterial/venous thromboembolism and myocardial infarct [53]. The data on Factor VIII discussed in this paper, although preliminary and limited in number, seem to suggest that the thrombogenicity of Sars-CoV2 infection might be linked to widespread endothelial damage. As previously described and confirmed in the present study, histological and immunohistochemical investigations revealed the presence of the virus within endothelial cells, promoting an inflammatory response. In light of this finding, pre-existing cardiovascular disease could be associated with adverse outcomes in COVID-19.

In this regard, more autopsies could have clarified whether different histological presentations are expressions of two chronologically subsequent phases of the natural history of the same pathology or represent two different pathways of manifestation of the same pathology. Indeed, as recently described by Ellinghaus et al., there is a gene cluster that can be linked with the patient’s response to the COVID-19 infection. Particularly, these authors found that the frequency of the risk allele of the lead variant at 3p21.31 (rs11385942) was higher among patients who were hospitalized in intensive care units [54]. In this way it would be very important to investigate the pro-coagulative substrate both in all subjects who died and in COVID-19 survivors [55]. For example, it would be very important to better explain the mortality rate of this virus, to genotype all subjects who died from COVID-19 for factor V Leiden (FVL) and prothrombin G20210A mutation (PTM),which are considered the two most common genetic polymorphisms known to increase the risks of thromboembolism [56,57,58,59,60].

In conclusion, it is important to perform more post-mortem investigations in order to clarify all aspects of the vascular hypothesis in the COVID-19 infection. As already suggested in February 2020 by Vetter et al. [61], if “COVID-19: A Puzzle with Many Missing Pieces” is true, there is no doubt that the missing piece is represented by autopsies. Without this piece, the puzzle is incomplete.

## Figures and Tables

**Table 1:** Main characteristics of the selected cases.

CASE	Sex/Age	Place of Death	Symptoms	Estimated Time from COVID-19 Infection	Laboratory Test for SARS-CoV-2 Using Quantitative RT-PCR (Allplex™ 2019 n-CoV Assay, Seegene, Seoul, Korea)
**1**	M 42 y	At home	Respiratory distress (no fever, no cough); two days after medical consultation he was found dead at home	4 days	Negative post-mortem nasopharyngeal and oropharyngeal flocked swabs. Positive to lung flocked swab.
**2**	M 70 y	In hospital	Fever, cough, asthenia without dyspnea; two days after medical consultation he died in hospital	12 days	Positive post-mortem nasopharyngeal flocked swab.
**CTL**	M 1 month	In hospital	Pneumonia, respiratory distress syndrome; he died in hospital	---	Negative post-mortem nasopharyngeal, oropharyngeal, and lung flocked swabs

**Table 2:** The main results of the immunohistochemical examination. The different force of immunohistochemical expression was encoded with the following legend: “negative” (−), “very weak” (+/−), “weak” (+), “moderate” (++), “strong” (+++), “very strong” (++++).

	Anti COVID-19	Anti CD-4	Anti CD-8	Anti CD-20	Anti CD-68	Anti CD-79	Anti ACE2	Anti FVIII	Anti IL6	Anti TNFα
CASE 1	++++	+	+++	+++	++++	++++	++++	++++	++	+++
CASE 2	++	+/−	+++	+++	++++	−	+++	++	++	+++
CTL	−	−	−	++	++++	+	+	−	+	++
